# Supplementary material for: Phosphatidylethanolamine N-Methyltransferase Knockout Modulates Metabolic Changes in Aging Mice
Source: Biomolecules. 2022 Sep 9;12(9):1270. doi: 10.3390/biom12091270 (PMC9496051; doi:10.3390/biom12091270)
Supplement: Supplementary file 1 [file biomolecules-12-01270-s001.zip › biomolecules-1877530-supplementary.pdf]

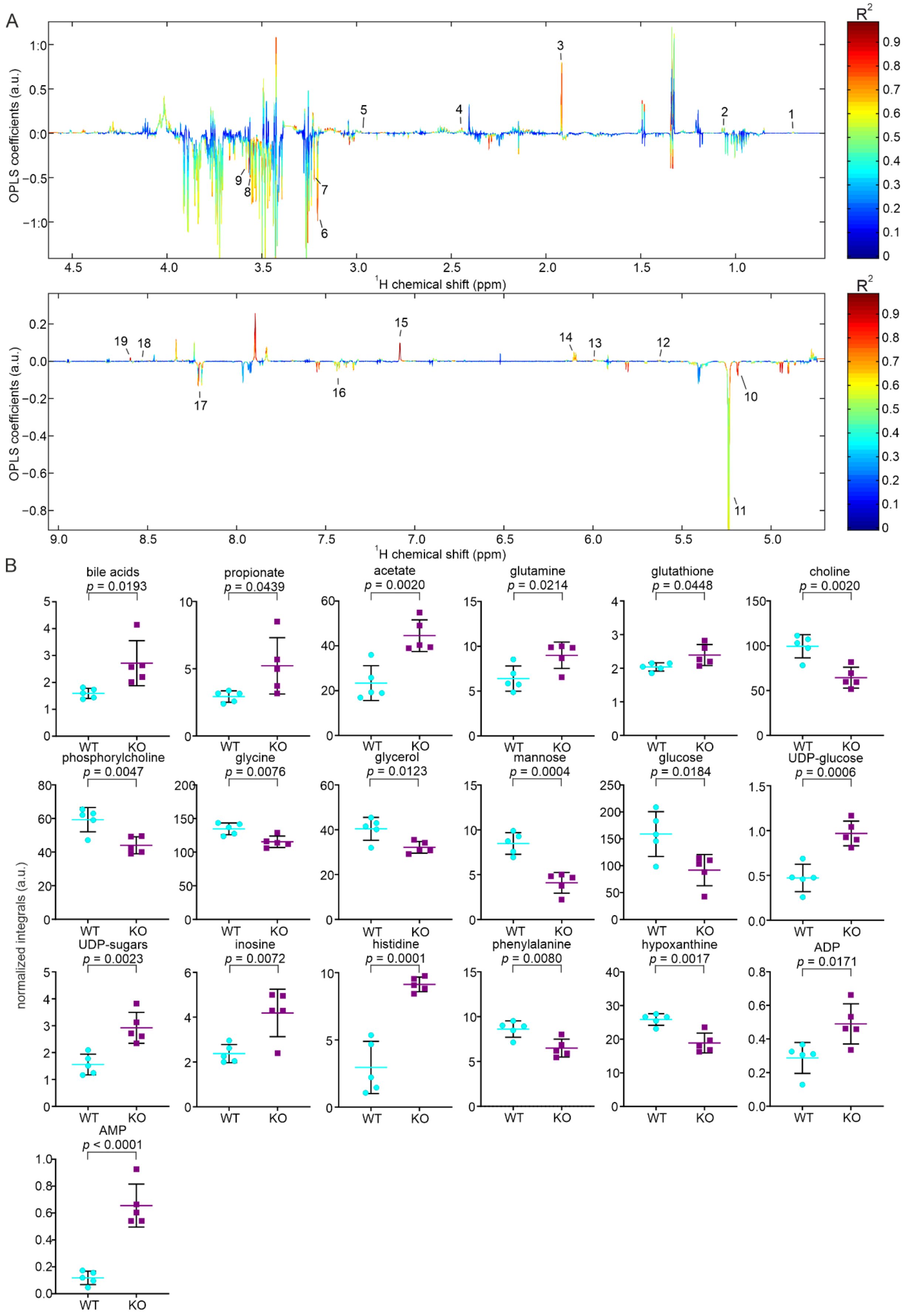

Figure S1. Altered metabolites from young liver samples. **(A)** Reduced spectra deduced based on ^{1}H NMR spectra of WT and PEMT KO liver (young mice) after normalization. Increased and decreased

signals found in the KO samples are pointing upward and downward, respectively. Labelled metabolites correspond to 1 bile acids, 2 propionate, 3 acetate, 4 glutamine, 5 glutathione, 6 choline, 7 phosphorylcholine, 8 glycine, 9 glycerol, 10 mannose, 11 glucose, 12 UDP-glucose, 13 UDP-sugars, 14 inosine, 15 histidine, 16 phenylalanine, 17 hypoxanthine, 18 ADP, 19 AMP. these metabolites are significantly altered ($p < 0.05$). **(B)** Plot of signal integration for labeled metabolite in (A), in supplementary figures S1-S13, mean, standard deviation (SD) and *p* value are deduced from Student's t-test as described in Materials and Methods.

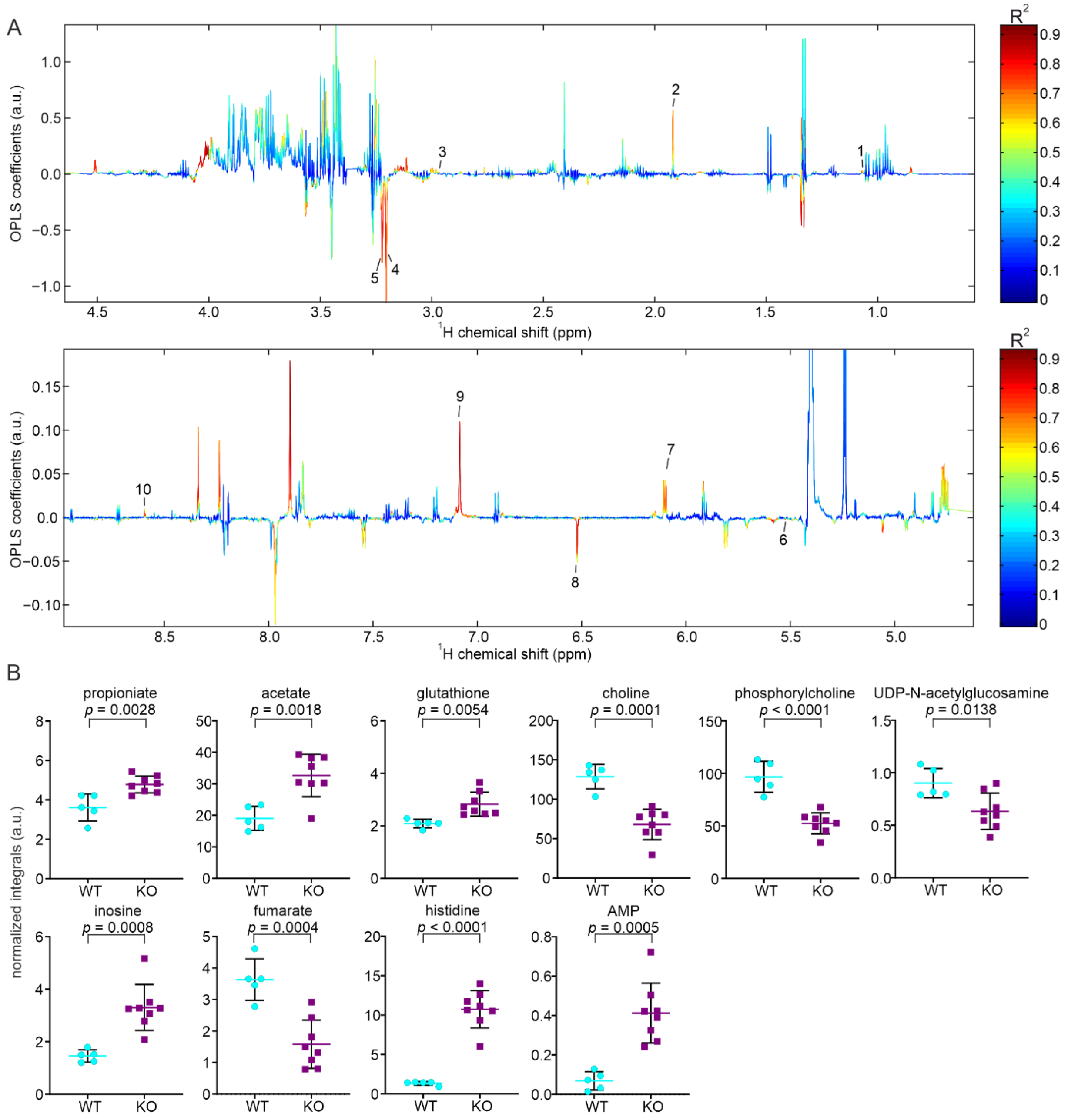

Figure S2. Altered metabolites from aged liver samples. **(A)** Reduced spectra deduced from ^{1}H NMR spectra of WT and PEMT KO liver (old mice) after normalization, with increased and decreased signals in the KO samples pointing upward and downward, respectively. Labelled metabolites correspond to significantly altered ($p < 0.05$) metabolites, i.e., 1 propionate, 2 acetate, 3 glutathione, 4 choline, 5 phosphorylcholine, 6 UDP-N-acetylglucosamine, 7 inosine, 8 fumarate, 9 histidine, 10 AMP. **(B)** Plot of signal integration for labeled metabolites in (A).

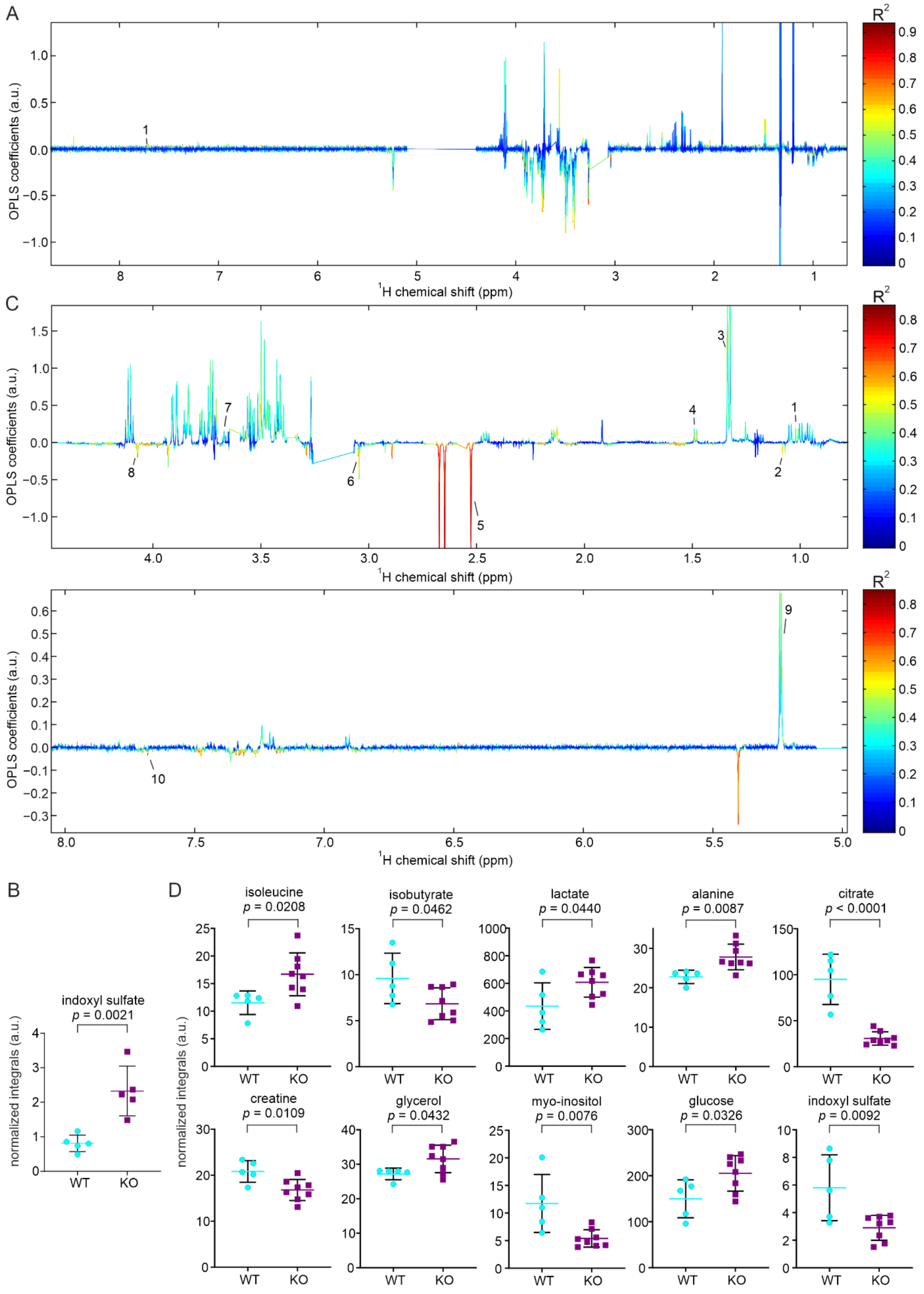

Figure S3. Altered metabolites from plasma samples of young and aged mice. **(A)** Reduced spectra deduced from ^{1}H NMR spectra of WT and PEMT KO plasma (young mice) after normalization. Increased and decreased signals in the KO samples are pointing upward and downward, respectively. Labelled metabolite 1 corresponds to indoxyl sulfate, which is significantly altered ($p < 0.05$). **(B)** Plots of signal

integrations for metabolites labeled in (A). **(C)** Reduced spectra deduced from ^{1}H NMR spectra of WT and PEMT KO plasma (old mice) after normalization. Increased and decreased signals in the KO samples are pointing upward and downward, respectively. Labelled metabolites correspond to significantly altered ($p < 0.05$) metabolites, i.e., 1 isoleucine, 2 isobutyrate, 3 lactate, 4 alanine, 5 citrate, 6 creatine, 7 glycerol, 8 myo-inositol, 9 glucose, 10 indoxyl sulfate. **(D)** Plots of signal integrations for metabolites labeled in (C).

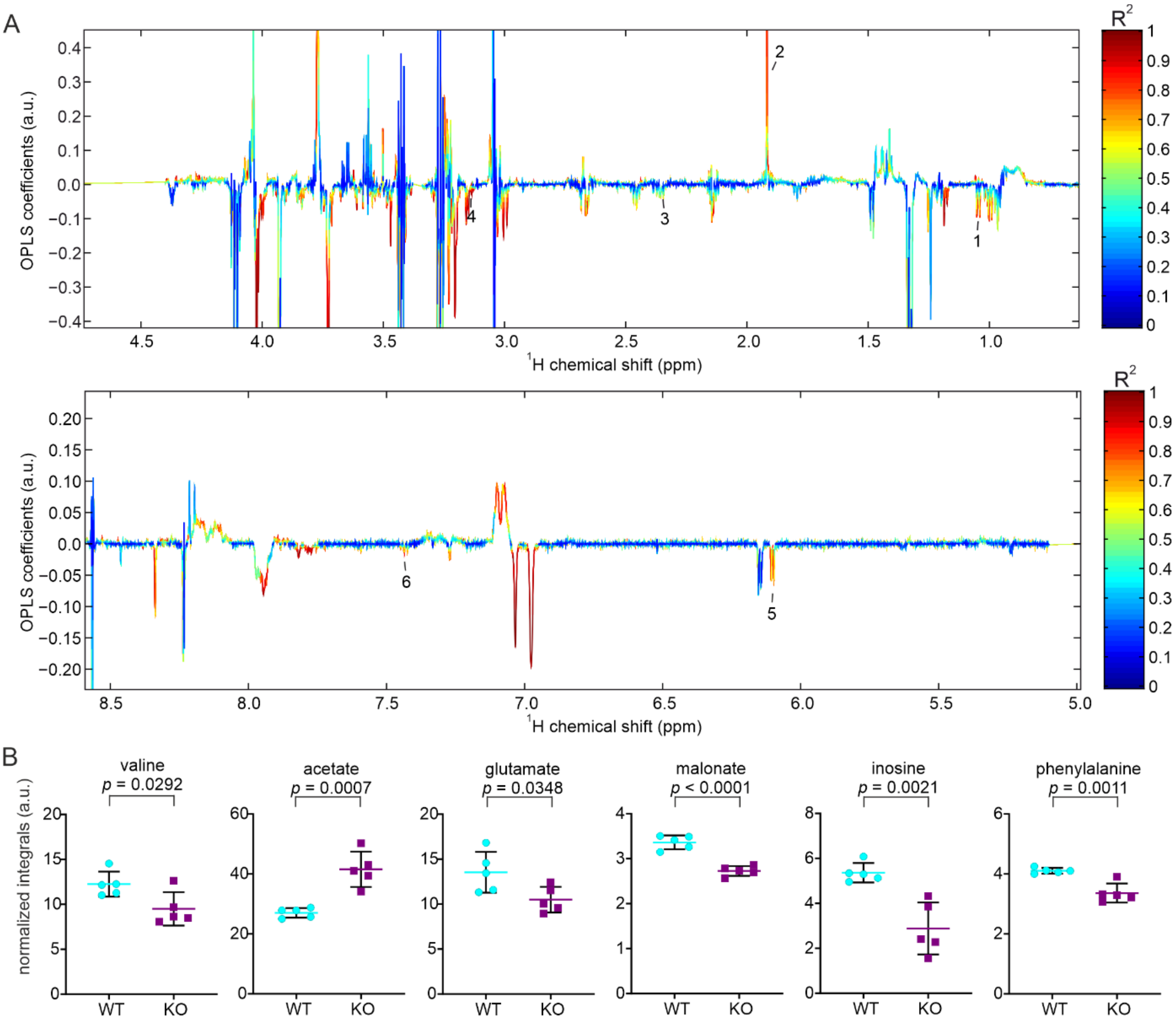

Figure S4. Altered metabolites from young skeletal muscle samples. **(A)** Reduced spectra deduced from ^{1}H NMR spectra of WT and PEMT KO skeletal muscle (young mice) after normalization. Increased and decreased signals in the KO samples are pointing upward and downward, respectively. Labelled metabolites correspond to significantly altered (p < 0.05) metabolites, i.e., 1 valine, 2 acetate, 3 glutamate, 4 malonate, 5 inosine, 6 phenylalanine. **(B)** Plots of signal integrations for metabolites labeled in (A).

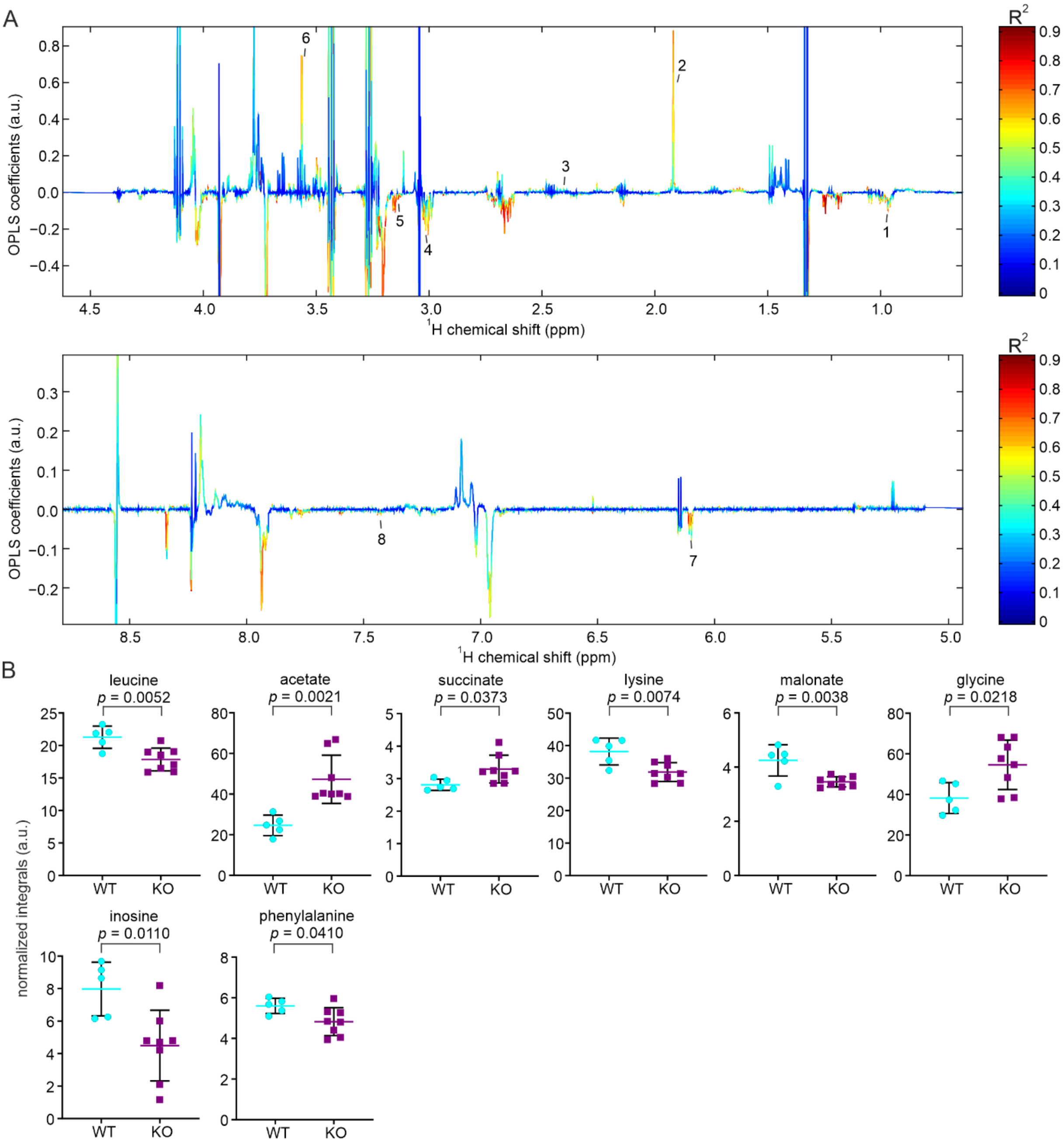

Figure S5. Altered metabolites from aged skeletal muscle samples. **(A)** Reduced spectra deduced from ^{1}H NMR spectra of WT and PEMT KO skeletal muscle (old mice) after normalization. Increased and decreased signals in the KO samples are pointing upward and downward, respectively. Labelled metabolites correspond to significantly altered ($p < 0.05$) metabolites, i.e., 1 leucine, 2 acetate, 3 succinate, 4 lysine, 5 malonate, 6 glycine, 7 inosine, 8 phenylalanine. **(B)** Plots of signal integrations for metabolites labeled in (A).

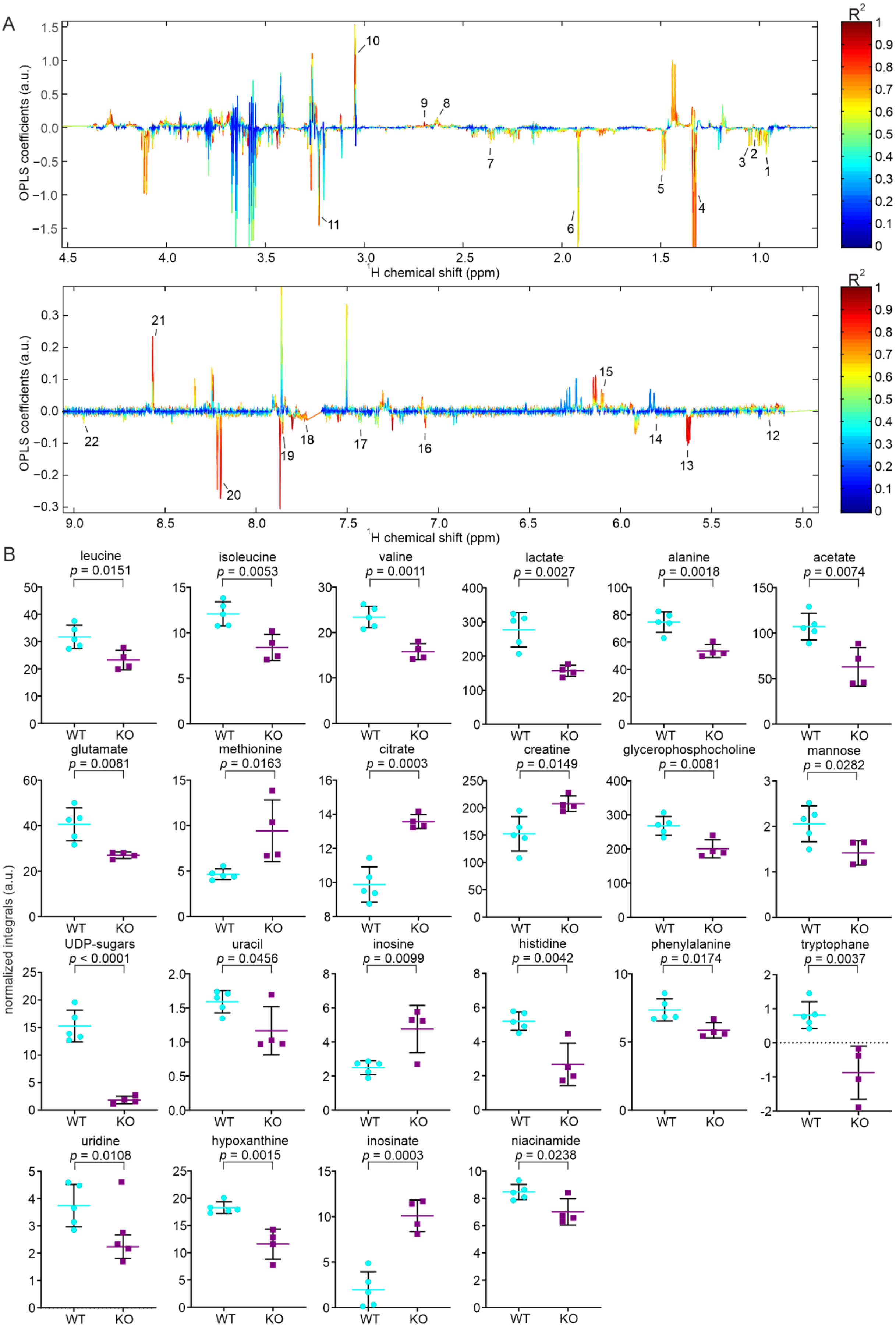

Figure S6. Altered metabolites from young BAT samples. **(A)** Reduced spectra deduced from ^{1}H NMR spectra of WT and PEMT KO BAT (young mice) after normalization. Increased and decreased signals in

the KO samples are pointing upward and downward, respectively. Labelled metabolites correspond to significantly altered ($p < 0.05$) metabolites, i.e., 1 leucine, 2 isoleucine, 3 valine, 4 lactate, 5 alanine, 6 acetate, 7 glutamate, 8 methionine, 9 citrate, 10 creatine, 11 glycerophosphocholine, 12 mannose, 13 UDP-sugars, 14 uracil, 15 inosine, 16 histidine, 17 phenylalanine, 18 tryptophan, 19 uridine, 20 hypoxanthine, 21 inosinate, 22 niacinamide. **(B)** Plots of signal integrations for metabolites labeled in (A).

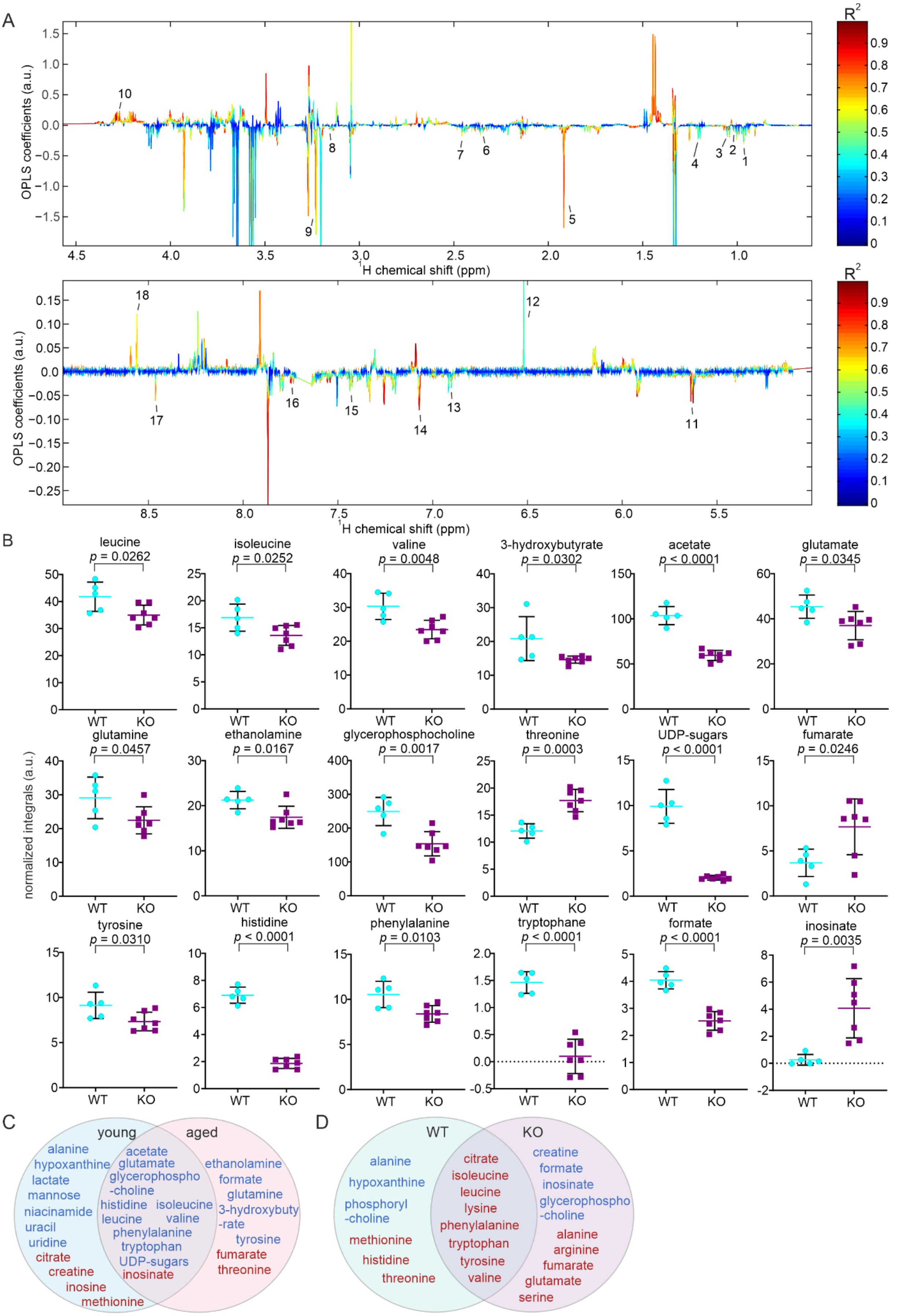

Figure S7. Altered metabolites from aged BAT samples. **(A)** Reduced spectra deduced from ^{1}H NMR spectra of WT and PEMT KO BAT (old mice) after normalization. Increased and decreased signals in the

KO samples are pointing upward and downward, respectively. Labelled metabolites correspond to significantly altered ($p < 0.05$) metabolites, i.e., 1 leucine, 2 isoleucine, 3 valine, 4 3-hydroxybutyrate, 5 acetate, 6 glutamate, 7 glutamine, 8 ethanolamine, 9 glycerophosphocholine, 10 threonine, 11 UDP-sugars, 12 fumarate, 13 tyrosine, 14 histidine, 15 phenylalanine, 16 tryptophan, 17 formate, 18 inosinate. **(B)** Plots of signal integrations for metabolites labeled in (A). **(C)** Venn diagram showing decreased (blue) and increased (red) metabolites in the young and aged PEMT KO BAT, corresponding to figure 3(A), 3(C), in the middle are the common metabolites. **(D)** Venn diagram showing aging biomarkers in the WT and KO BAT, in the middle are the common metabolites.

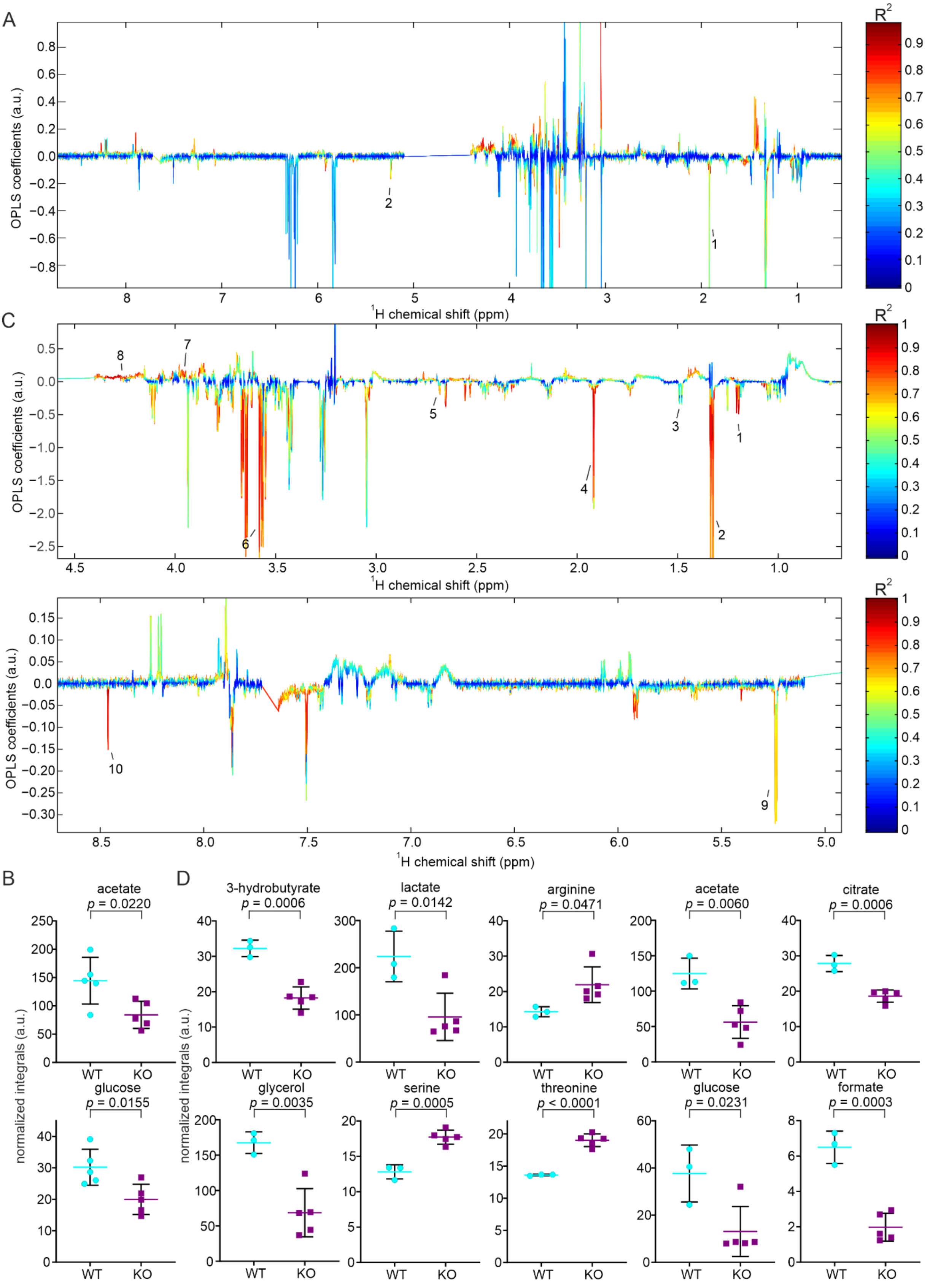

Figure S8. Altered metabolites from young and aged WAT samples. **(A)** Reduced spectra deduced from ^{1}H NMR spectra of WT and PEMT KO WAT (young mice) after normalization. Increased and decreased signals in the KO samples are pointing upward and downward, respectively. Labelled metabolites correspond to significantly altered ($p < 0.05$) metabolites, i.e., 1 acetate, 2 glucose. **(B)** Plot of signal integration for labeled metabolites in (A). **(C)** Reduced spectra deduced from ^{1}H NMR spectra of WT

and PEMT KO WAT (old mice) after normalization. Increased and decreased signals in the KO samples are pointing upward and downward, respectively. Labelled metabolites correspond to significantly altered ($p < 0.05$) metabolites, i.e., 1 3-hydroxybutyrate, 2 lactate, 3 arginine, 4 acetate, 5 citrate, 6 glycerol, 7 serine, 8 threonine, 9 glucose, 10 formate. **(D)** Plots of signal integrations for metabolites labeled in (C).

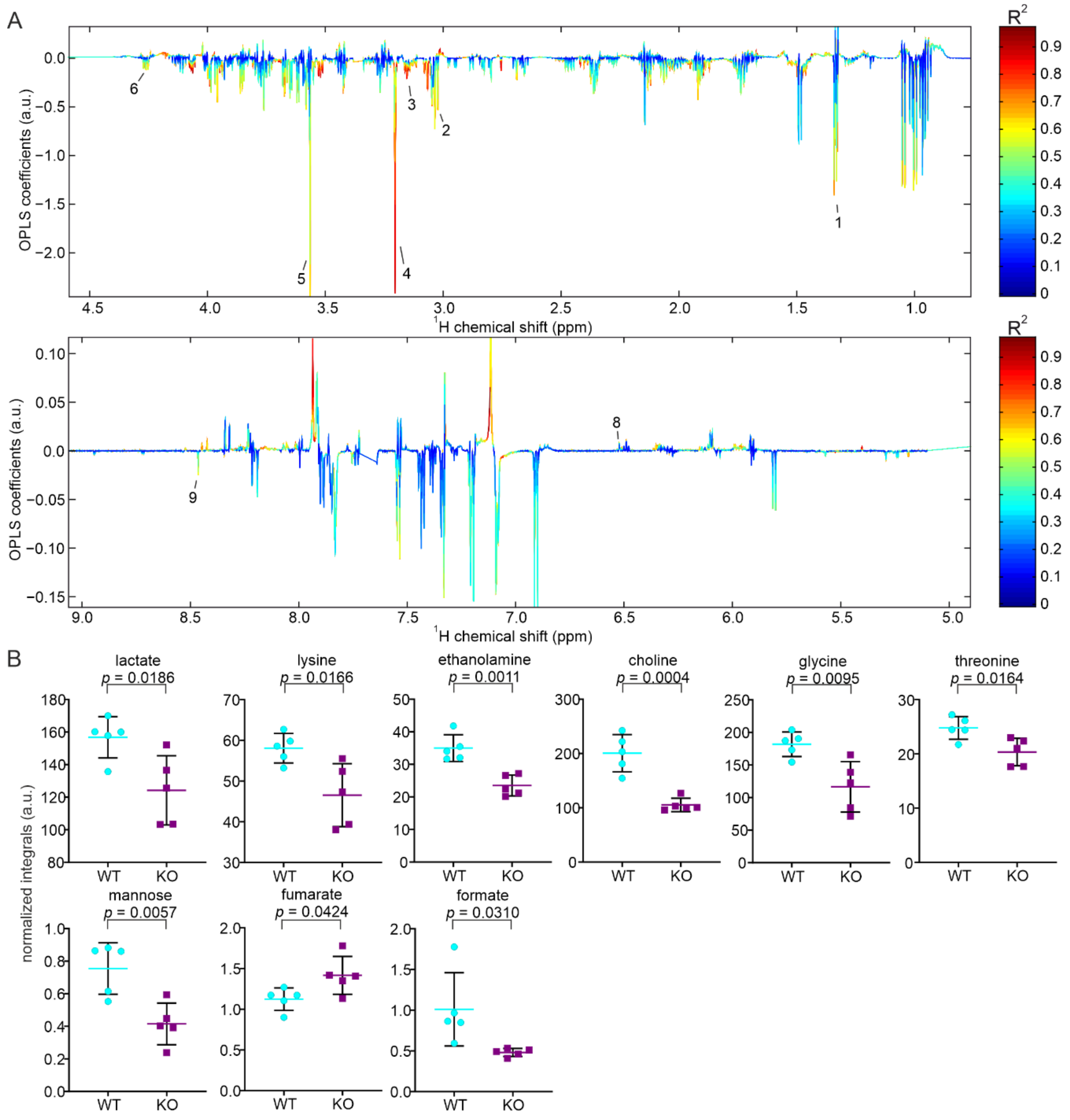

Figure S9. Altered metabolites from young duodenum samples. **(A)** Reduced spectra deduced from ^{1}H NMR spectra of WT and PEMT KO duodenum (young mice) after normalization. Increased and decreased signals in the KO samples are pointing upward and downward, respectively. Labelled metabolites correspond to significantly altered (p < 0.05) metabolites, i.e., 1 lactate, 2 lysine, 3 ethanolamine, 4 choline, 5 glycine, 6 threonine, 7 mannose, 8 fumarate, 9 formate. **(B)** Plots of signal integrations for metabolites labeled in (A).

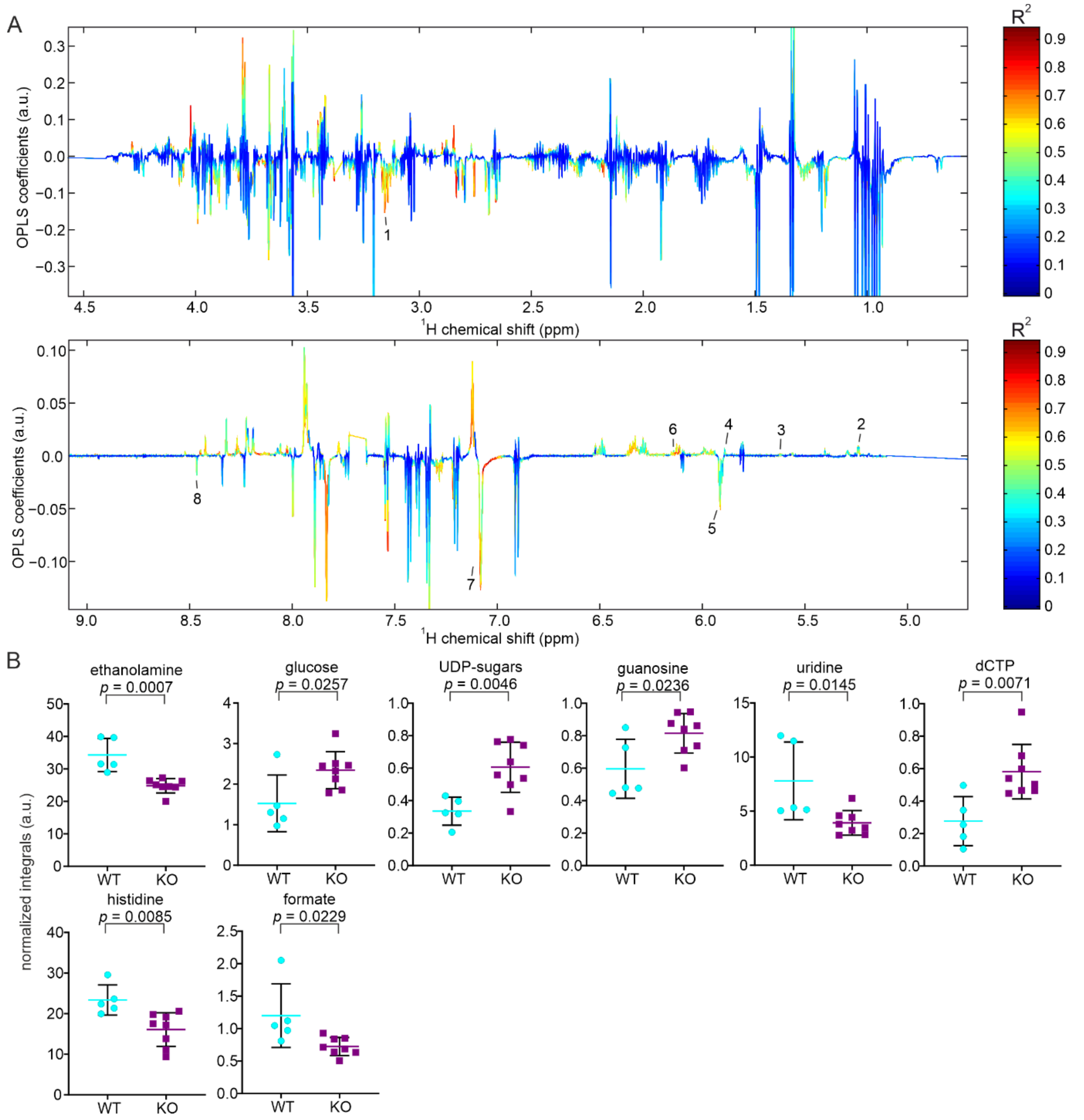

Figure S10. Altered metabolites from aged duodenum samples. **(A)** Reduced spectra deduced from ^{1}H NMR spectra of WT and PEMT KO duodenum (old mice) after normalization. Increased and decreased signals in the KO samples are pointing upward and downward, respectively. Labelled metabolites correspond to significantly altered ($p < 0.05$) metabolites, i.e., 1 ethanolamine, 2 glucose, 3 UDP-sugars, 4 guanosine, 5 uridine, 6 dCTP, 7 histidine, 8 formate. **(B)** Plots of signal integrations for metabolites labeled in (A).

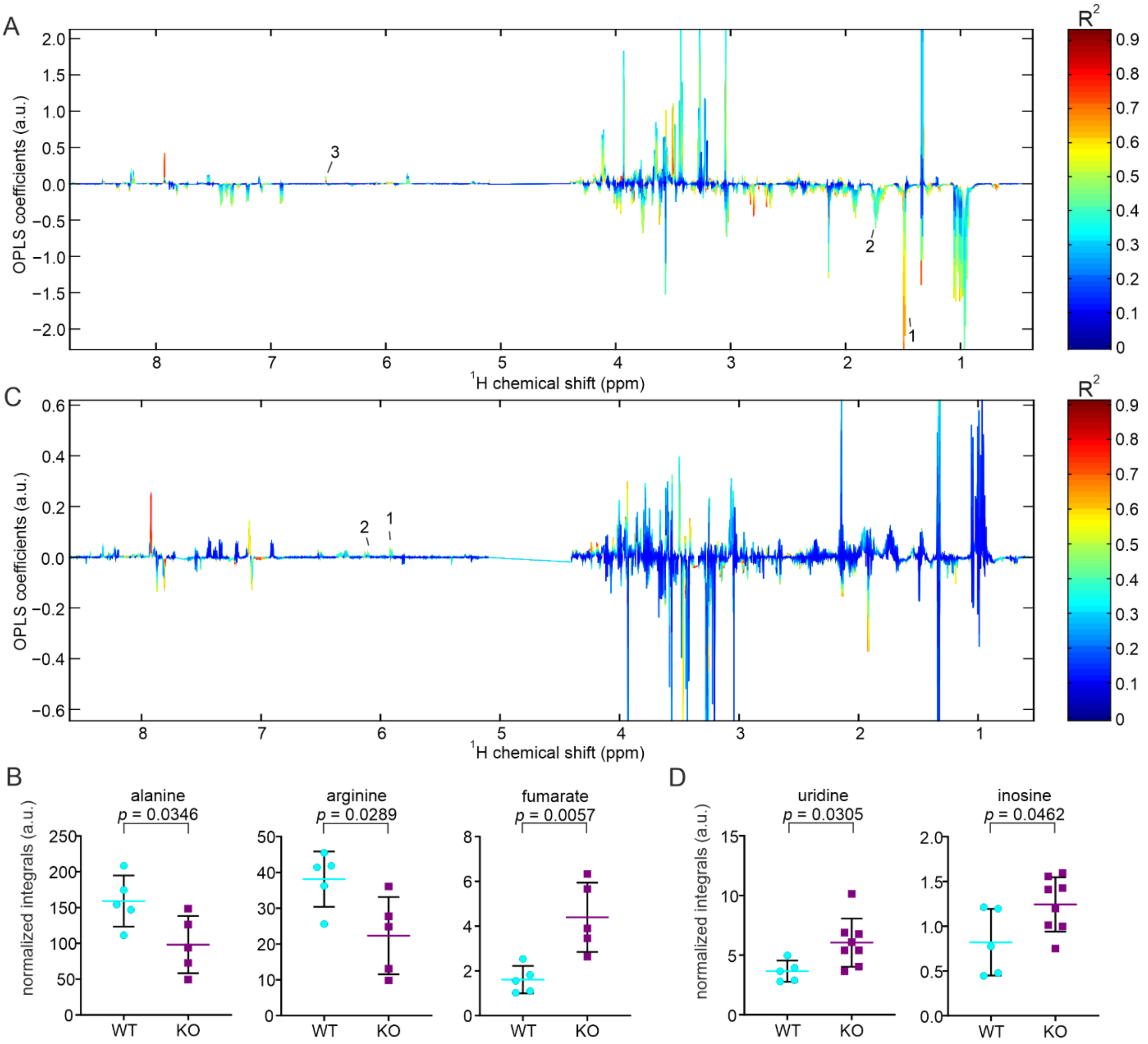

Figure S11. Altered metabolites from young and aged jejunum samples. **(A)** Reduced spectra deduced from ^{1}H NMR spectra of WT and PEMT KO jejunum (young mice) after normalization. Increased and decreased signals in the KO samples are pointing upward and downward, respectively. Labelled metabolites correspond to significantly altered (p < 0.05) metabolites, i.e., 1 alanine, 2 arginine, 3 fumarate. **(B)** Plot of signal integration for labeled metabolites in (A). **(C)** Reduced spectra deduced from ^{1}H NMR spectra of WT and PEMT KO jejunum (old mice) after normalization. Increased and decreased signals in the KO samples are pointing upward and downward, respectively. Labelled metabolites correspond to significantly altered (p < 0.05) metabolites, i.e., 1 uridine, 2 inosine. **(D)** Plots of signal integrations for metabolites labeled in (A).

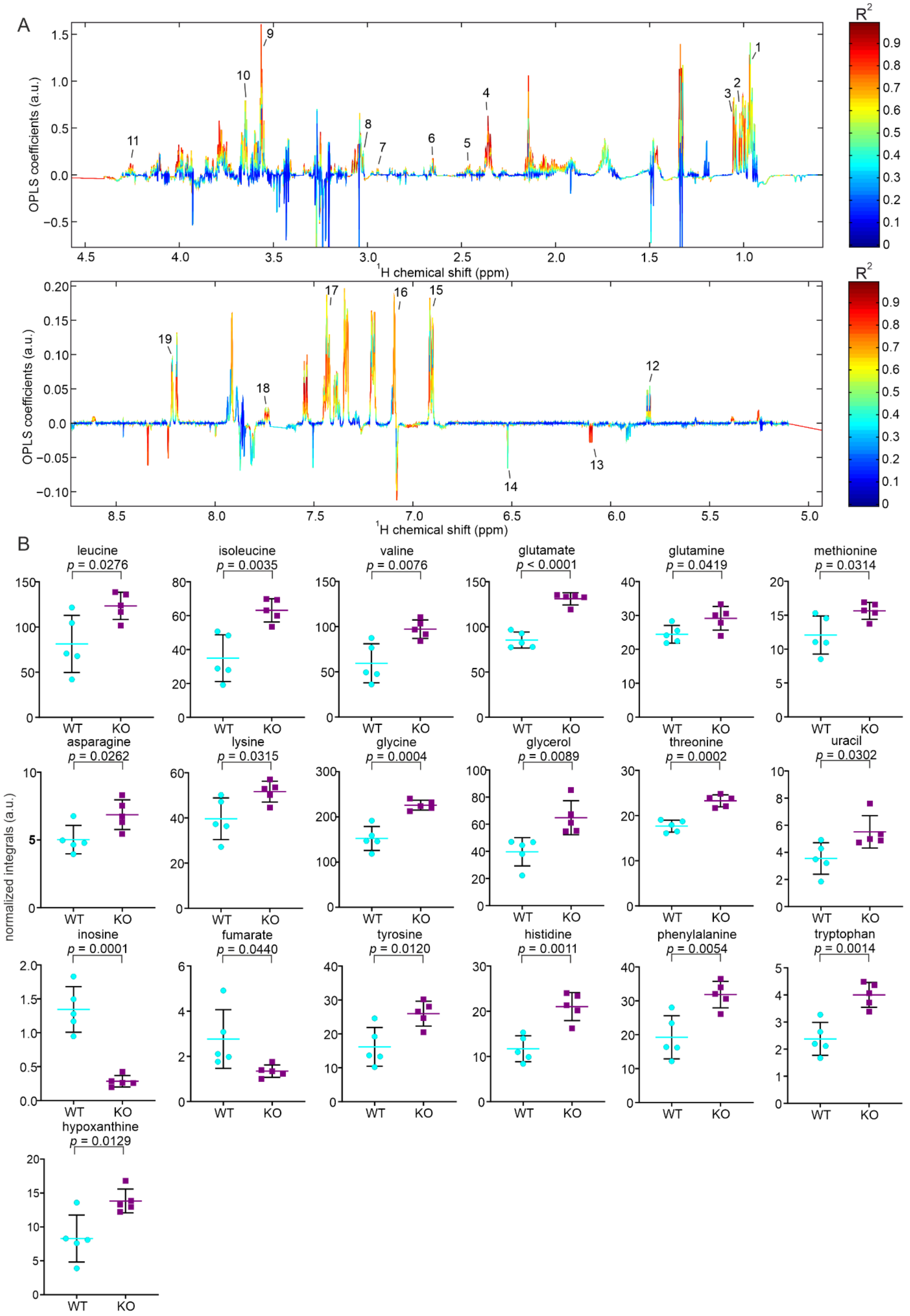

Figure S12. Altered metabolites from young ileum samples. **(A)** Reduced spectra deduced from ^{1}H NMR spectra of WT and PEMT KO ileum (young mice) after normalization. Increased and decreased signals in the KO samples are pointing upward and downward, respectively. Labelled metabolites correspond

to significantly altered ($p < 0.05$) metabolites, i.e., 1 leucine, 2 isoleucine, 3 valine, 4 glutamate, 5 glutamine, 6 methionine, 7 asparagine, 8 lysine, 9 glycine, 10 glycerol, 11 threonine, 12 uracil, 13 inosine, 14 fumarate, 15 tyrosine, 16 histidine, 17 phenylalanine, 18 tryptophan, 19 hypoxanthine. **(B)** Plots of signal integrations for metabolites labeled in (A).

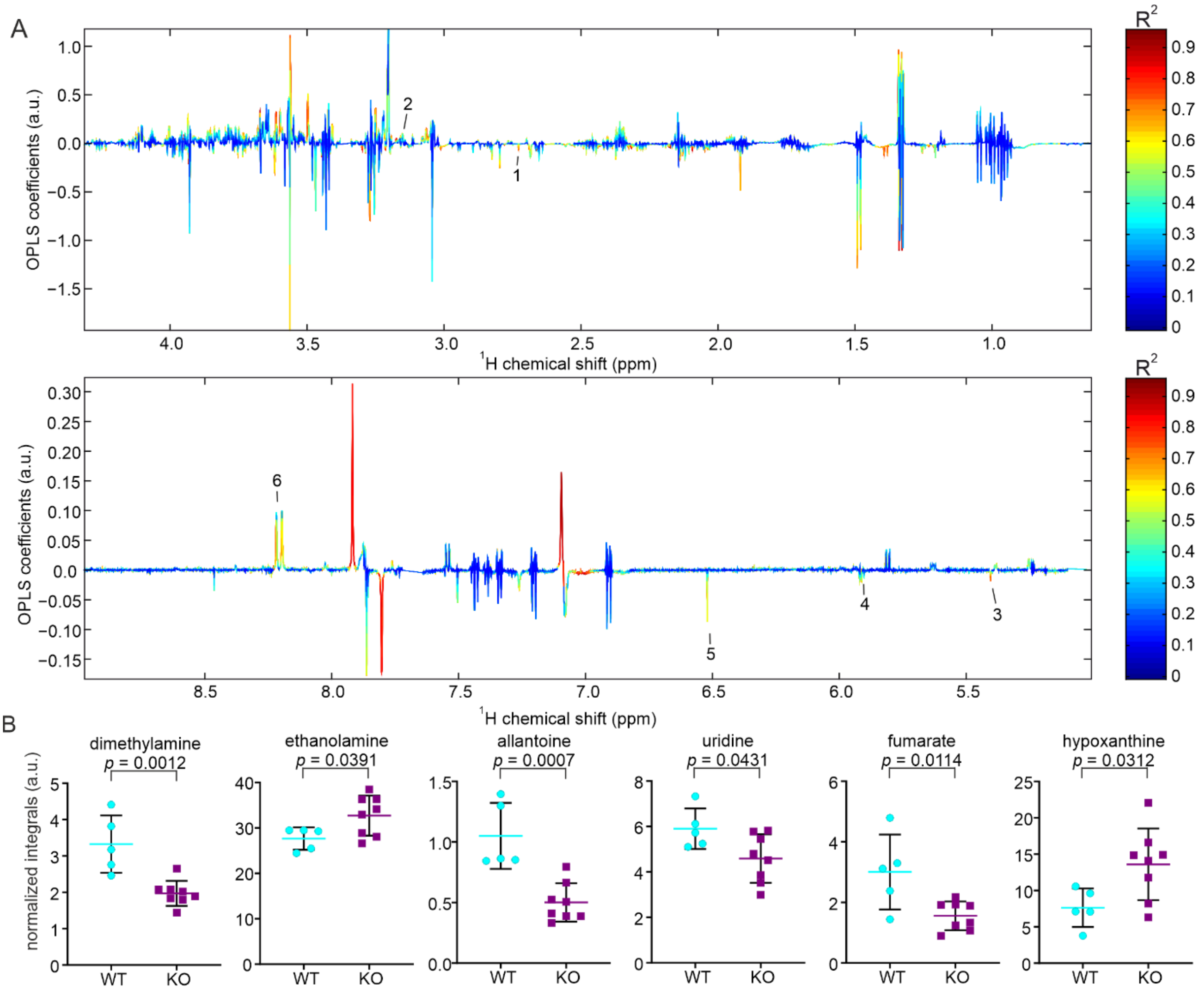

Figure S13. Altered metabolites from aged ileum samples. **(A)** Reduced spectra deduced from ^{1}H NMR spectra of WT and PEMT KO ileum (old mice) after normalization. Increased and decreased signals in the KO samples are pointing upward and downward, respectively. Labelled metabolites correspond to significantly altered (p < 0.05) metabolites, i.e., 1 dimethylamine, 2 ethanolamine, 3 allantoine, 4 uridine, 5 fumarate, 6 hypoxanthine. **(B)** Plots of signal integrations for metabolites labeled in (A).

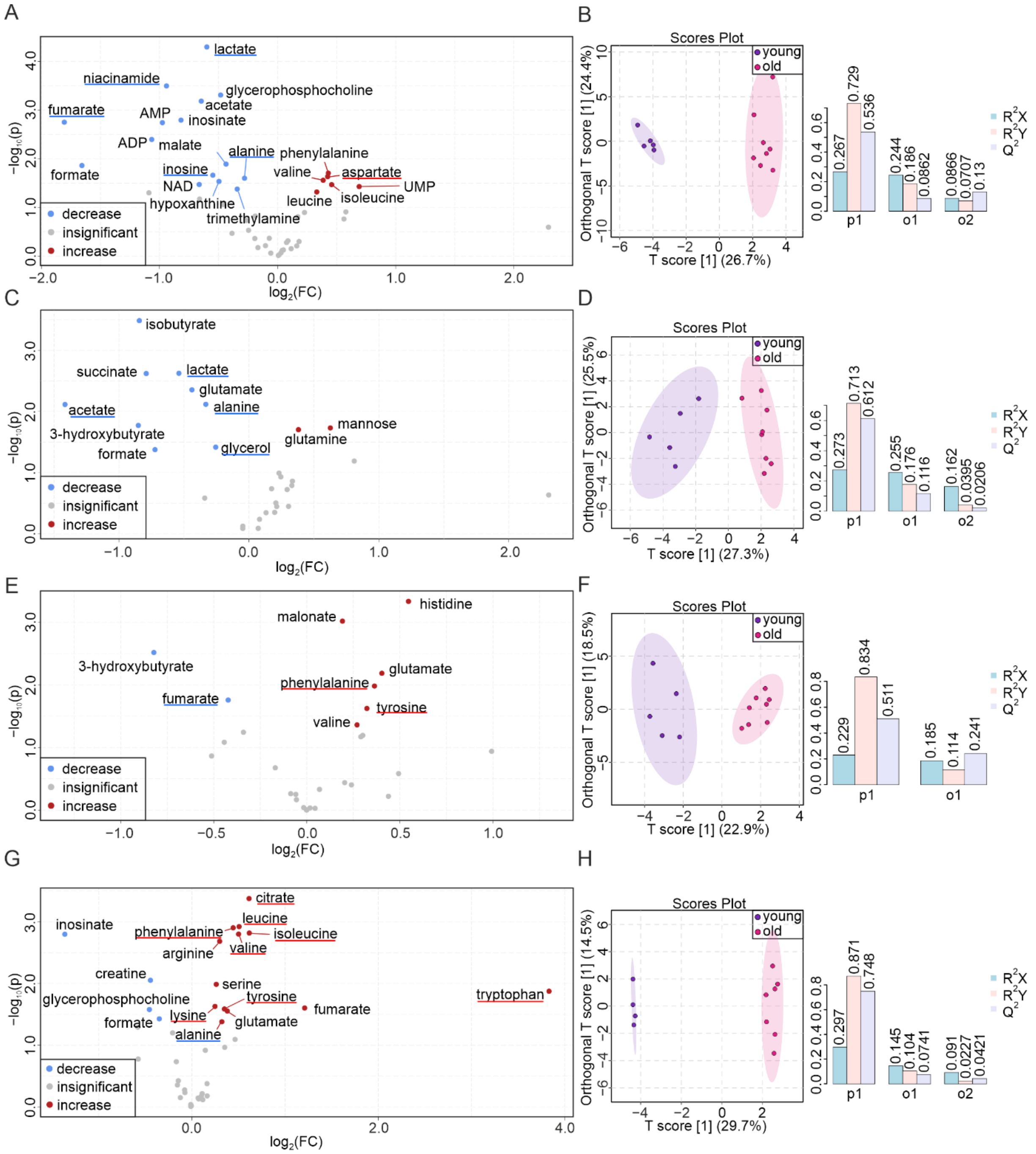

Figure S14. Volcano plot and statistical analysis of the aging metabolome from PEMT KO tissues (part one). **(A)** Volcano plot showing up- and down-regulated metabolites in the aging liver of PEMT KO mice, indicated by red and blue dots, respectively. All grey dots correspond to metabolites with insignificant changes in this and the next figure. **(B)** O-PLS-DA plot showing the two clusters of liver samples corresponding to young (purple) and old (pink) PEMT KO mice, along with the cross-validation parameters R^2X, R^2Y and Q^2 of the first predictive component (p1) and the two orthogonal components (o1, o2). **(C)** Volcano plot indicating up- and down-regulated metabolites (red and blue dots, respectively) in the aging plasma of PEMT KO mice. **(D)** O-PLS-DA plot showing the two clusters of plasma from young (purple) and old (pink) PEMT KO mice, along with R^2X, R^2Y and Q^2 of p1, o1 and o2. **(E)** Volcano plot showing up- and down-regulated metabolites (indicated by red and blue dots, respectively) in the aging skeletal muscle of PEMT KO mice. **(F)** O-PLS-DA plot showing the two clusters of skeletal muscle from young (purple) and old (pink) PEMT KO mice, along with R^2X, R^2Y and Q^2 of p1 and o1. **(G)** Volcano plot indicating up(red)- and down(blue)-regulated metabolites from aging BAT of

PEMT KO mice. **(H)** O-PLS-DA plot showing the two clusters of PEMT KO BAT corresponding to young (purple) and old (pink) mice, along with R^2X, R^2Y and Q^2 of p1, o1 and o2. Aging biomarkers which have also been found in the liver, plasma, skeletal muscle and BAT of WT mice are labelled with underlines (blue and red correspond to those with decreased and increased concentrations, respectively).

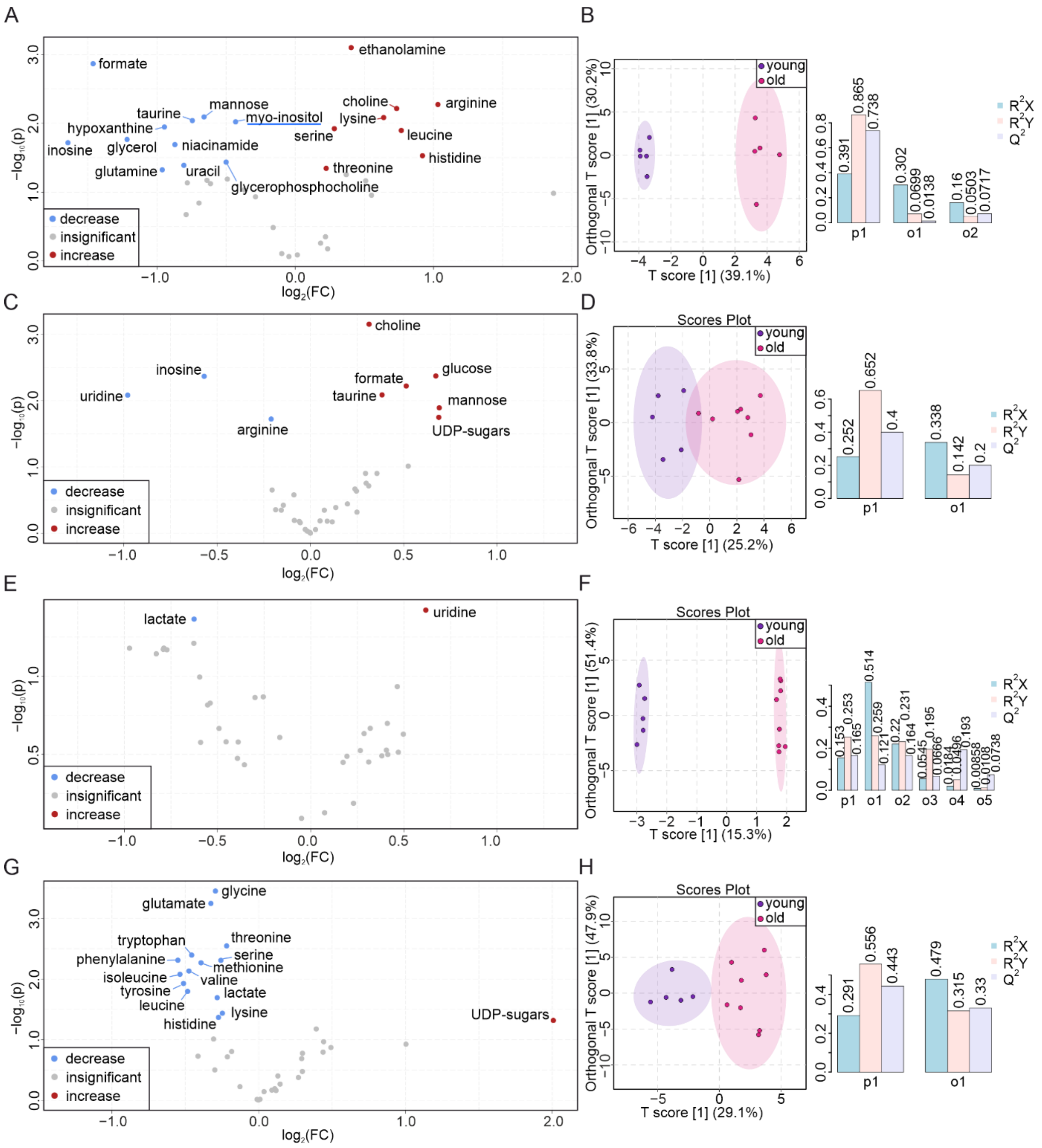

Figure S15. Volcano plot and statistical analysis of the aging metabolome from PEMT KO tissues (part two). **(A)** Volcano plot showing increased (red) and decreased (blue) metabolites in the aging WAT of PEMT KO mice. **(B)** O-PLS-DA plot demonstrate the discriminant clustering of WAT from young (purple) and old (pink) PEMT KO mice, along with R^2X, R^2Y and Q^2 of p1, o1 and o2. **(C)** Volcano plot (left) corresponding to increased (red) and decreased (blue) metabolites in the aged duodena of PEMT KO mice. **(D)** O-PLS-DA showing two clusters of duodena of young (purple) and old (pink) PEMT KO mice, along with R^2X, R^2Y and Q^2 of p1 and o1. **(E)** Volcano plot showing up- and down-regulated metabolites (red and blue dots, respectively) in aged jejuna of PEMT KO mice. **(F)** Discriminant clustering of jejuna from young (purple) and old (pink) PEMT KO mice on the O-PLS-DA plot, with R^2X, R^2Y and Q^2 of p1 and the five orthogonal components (o1-o5). **(G)** Increased (red) and decreased (blue) metabolites in the aged ilea of PEMT KO mice on volcano plot. **(H)** O-PLS-DA plot of ilea from young (purple) and old (pink) PEMT KO mice, along with R^2X, R^2Y and Q^2 of p1 and o1. Aging biomarkers which have also been found in the WT WAT, duodena, jejuna and ilea are labelled with underlines (blue and red correspond to those with decreased and increased concentrations, respectively).

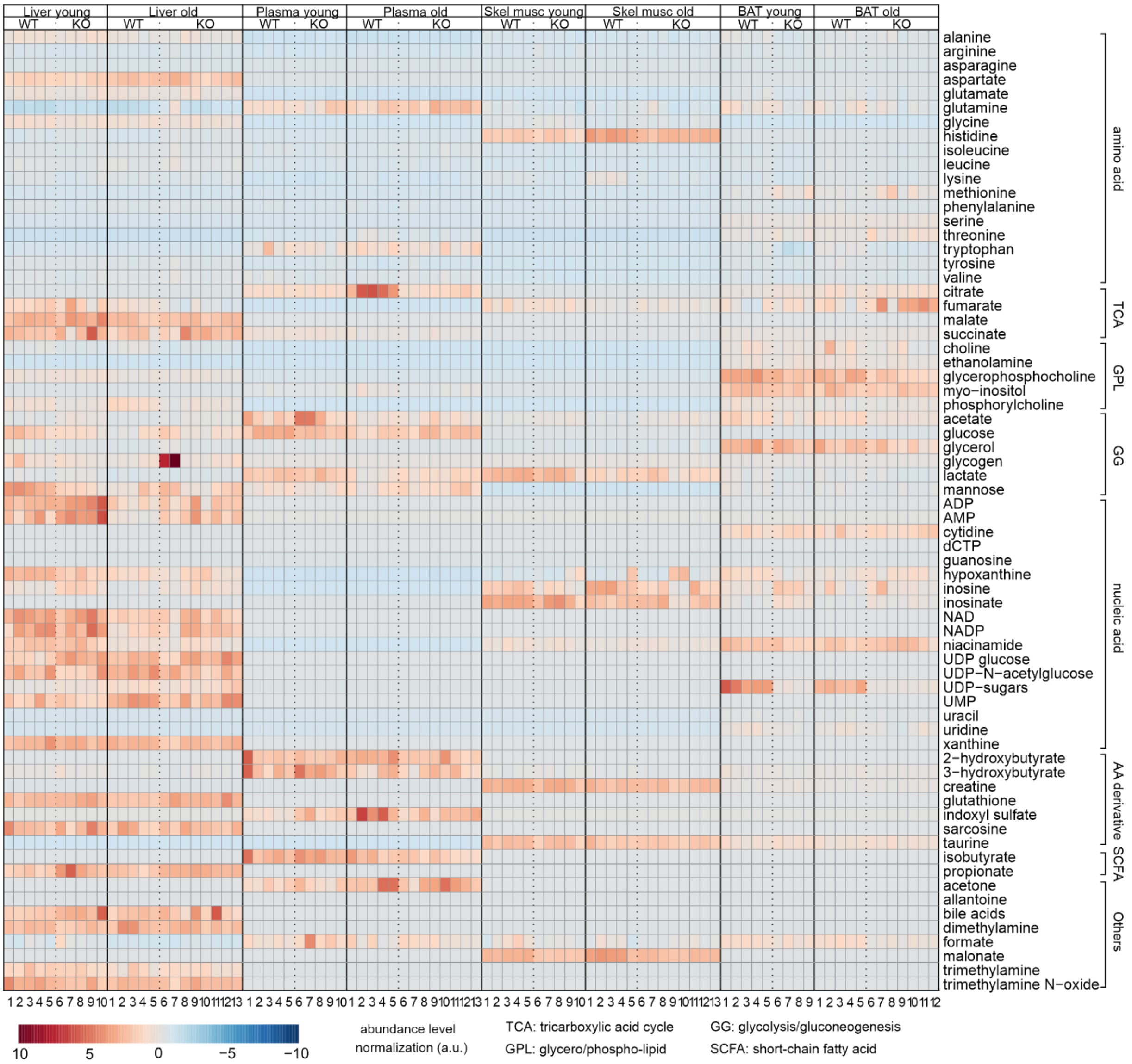

Figure S16. Heatmap of the quantified metabolites in liver, plasma, skeletal muscle, and BAT. The respective tissues, age of mice, and genotypes are indicated on top, metabolites at right. Sample numbers for each pairwise comparison is given below (young, n = 9-10; old, n = 8-13). Each single cell represents a relative abundance of the corresponding metabolite compared to the average. Within the same tissue data from all 4 groups are normalized to enable the comparison, from which metabolites at higher concentrations are in red, metabolites at lower concentrations are in blue.

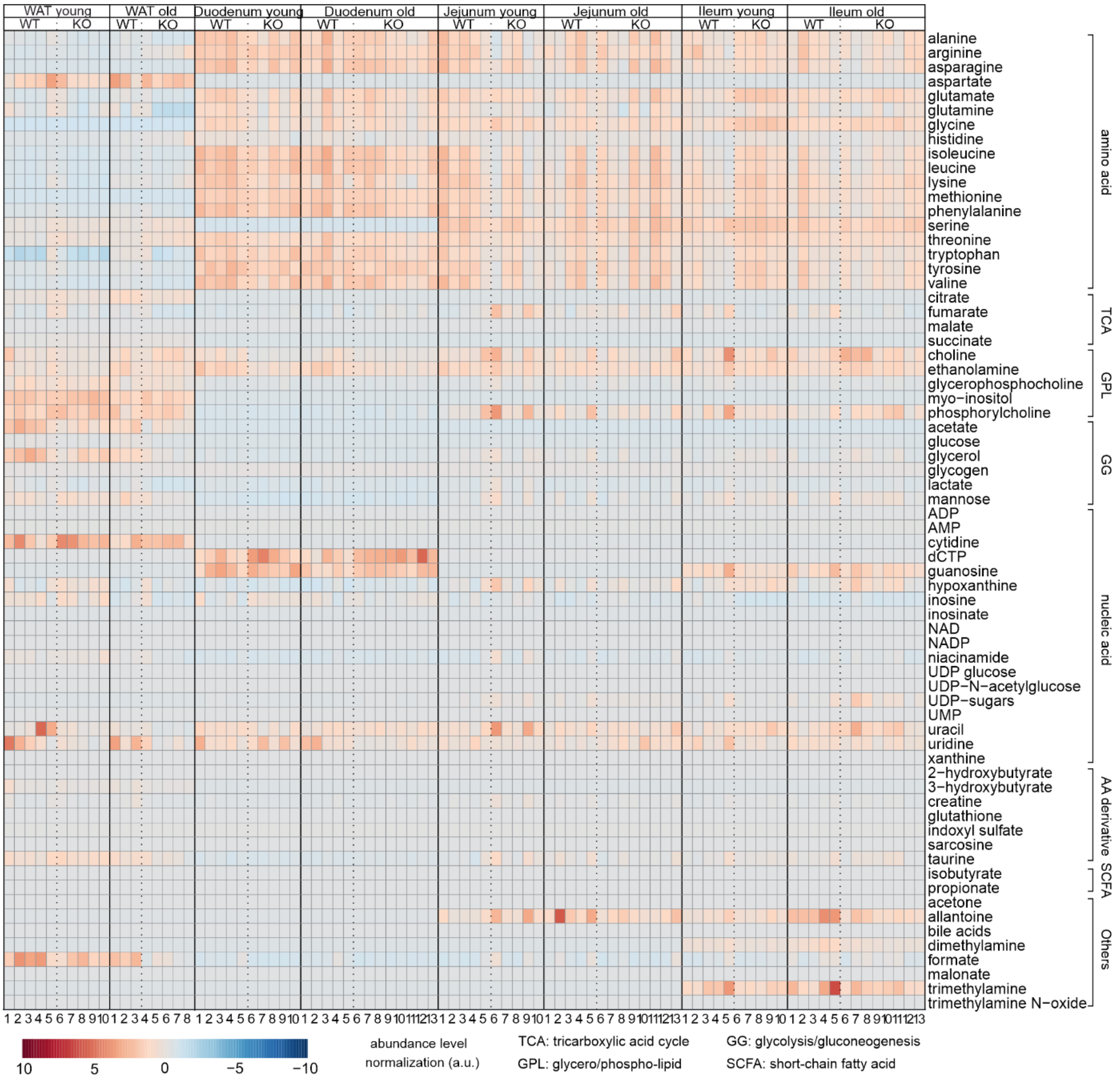

Figure S17. Heatmap of metabolites identified in WAT, duodenum, jejunum, and ileum. The respective tissues, age of mice, and genotypes are indicated on the top, metabolites on the right. Sample numbers are given below. Metabolites of higher and lower concentrations are in red and blue, respectively.

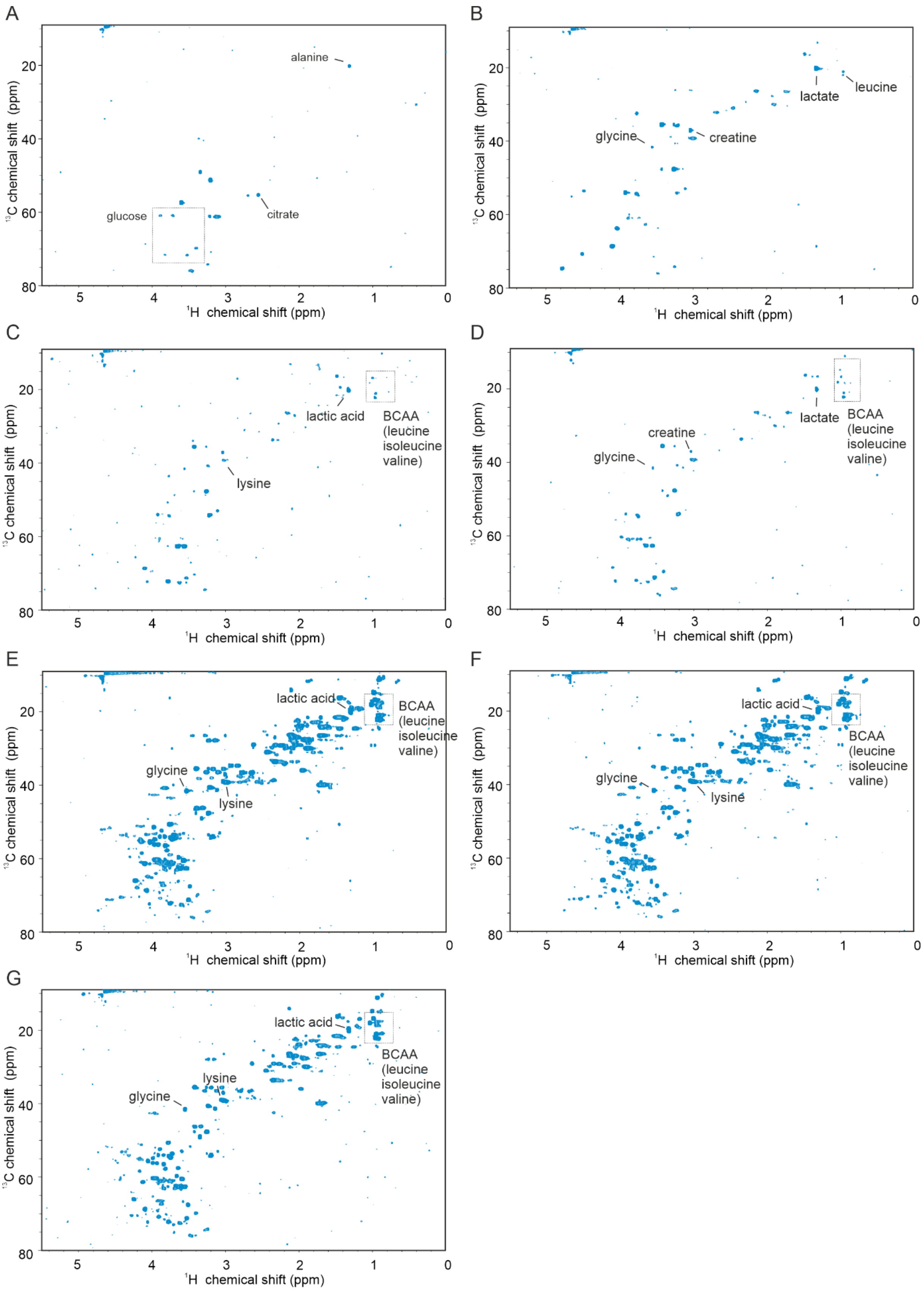

Figure S18. 2D ^{1}H-^{13}C HSQC NMR spectra showing metabolome from one of the PEMT KO samples. Each spectrum corresponds to (A) plasma, (B) skeletal muscle, (C) BAT, (D) WAT, (E) duodenum, (F) jejunum, and (G) ileum.
